# The Binge Eating Genetics Initiative (BEGIN): study protocol

**DOI:** 10.1186/s12888-020-02698-7

**Published:** 2020-06-16

**Authors:** Cynthia M. Bulik, Jonathan E. Butner, Jenna Tregarthen, Laura M. Thornton, Rachael E. Flatt, Tosha Smith, Ian M. Carroll, Brian R.W. Baucom, Pascal R. Deboeck

**Affiliations:** 1 Department of Psychiatry, University of North Carolina at Chapel Hill, CB #7160, 101 Manning Drive, Chapel Hill, NC 27599-7160 USA; 2 Department of Nutrition, University of North Carolina at Chapel Hill, Chapel Hill, NC USA; 3 Department of Medical Epidemiology and Biostatistics, Karolinska Institutet, Stockholm, Sweden; 4 Department of Psychology, University of Utah, Salt Lake City, UT USA; 5 Recovery Record, San Francisco, CA USA; 6 Department of Psychology and Neuroscience, University of North Carolina at Chapel Hill, Chapel Hill, NC USA

**Keywords:** Binge eating, Eating disorders, Wearable technology, Mobile application, Genetics, Microbiome

## Abstract

**Background:**

The Binge Eating Genetics Initiative (BEGIN) is a multipronged investigation examining the interplay of genomic, gut microbiota, and behavioral factors in bulimia nervosa and binge-eating disorder.

**Methods:**

1000 individuals who meet current diagnostic criteria for bulimia nervosa or binge-eating disorder are being recruited to collect saliva samples for genotyping, fecal sampling for microbiota characterization, and recording of 30 days of passive data and behavioral phenotyping related to eating disorders using the app *Recovery Record* adapted for the Apple Watch.

**Discussion:**

BEGIN examines the interplay of genomic, gut microbiota, and behavioral factors to explore etiology and develop predictors of risk, course of illness, and response to treatment in bulimia nervosa and binge-eating disorder. We will optimize the richness and longitudinal structure of deep passive and active phenotypic data to lay the foundation for a personalized precision medicine approach enabling just-in-time interventions that will allow individuals to disrupt eating disorder behaviors in real time before they occur.

**Trial registration:**

The ClinicalTrials.gov identifier is NCT04162574. November 14, 2019, Retrospectively Registered.

## Background

Bulimia nervosa (BN: lifetime prevalence of 1.5% in women and 0.5% in men) and binge-eating disorder (BED: lifetime prevalence of 3.5% in women and 2% in men) are common debilitating eating disorders [1]. BN is marked by uncontrollable eating episodes coupled with compensatory behaviors, whereas BED includes similarly defined binge episodes only in the absence of regular compensatory behaviors. Both disorders are highly heritable (41–82% [2–7]), carry high psychiatric and somatic comorbidity, and have high medication and healthcare utilization, whether or not comorbid obesity is present [8–11]. Suicide risk is significantly elevated in both disorders [12].

The Binge Eating Genetics Initiative (BEGIN) is a multipronged research study that 1) examines the interplay of genomic, gut microbiota, and behavioral factors to explore etiology and develop predictors of risk, course of illness, and response to treatment in BN and BED; and 2) optimizes the richness and longitudinal structure of deep passive and active phenotypic data to lay the foundation for a personalized precision medicine approach enabling just-in-time interventions that will allow individuals to disrupt eating disorder behaviors in real time before they occur.

### Genomics

Despite their prevalence and the attendant personal and social costs, research into the genetic underpinnings of BN and BED is essentially absent. BEGIN represents the first contribution to a global effort to amass an adequate sample size to conduct a genome-wide association study of BN and BED in collaboration with the Eating Disorders Working Group of the Psychiatric Genomics Consortium (PGC-ED). The PGC-ED has rapidly advanced the study of the genomics of anorexia nervosa [13, 14] identifying eight significant loci and reporting a panel of genetic correlations suggesting that anorexia nervosa may have both psychiatric and metabolic etiological underpinnings. BEGIN will further the mission of the PGC-ED by launching a parallel investigation into BN and BED.

### Intestinal microbiota

Inspired by reports of associations between enteric microbes, host metabolism, and host behavior [15–17] along with reported differences between gut microbiota composition from patients with anorexia nervosa and healthy individuals [18–25], we have incorporated the study of the intestinal microbiota into BEGIN. In addition to characterizing the biogeography of the human microbiome (cumulative genomes of the microbiota) of BEGIN participants, our analyses will identify associations between genes and microbial composition in BN/BED. The intention is to better understand the biological mechanisms of these illnesses in an effort to help identify potential drug targets and opportunities for novel interventions.

### Deep phenotyping

We are capturing real-time longitudinal digital phenotypic data on individuals with BN/BED that reflect the true complexity of human behavior. Using Apple Watch and iPhone devices, we are collecting *active* data on binge-eating, purging, nutrition, mood, and cognitions with a widely-used cognitive-behavioral based eating disorder app *Recovery Record* [26] and *passive* sensor data via native applications collected over a 30-day period. We will combine active *Recovery Record*-based measures and passively collected, continuous, sensor-based measurements of autonomic nervous system (ANS) activity and actigraphy to characterize patterns of when and where individuals are more/less likely to binge and/or purge in their daily lives. Finally, across and within individuals, we will identify low-risk and high-risk passive data patterns that will facilitate the prediction of transitions to high risk states signaling impending binge or purge episodes (time-stamped by active app monitoring). This work has the potential to transform the standard of care for BN and BED by transcending current cognitive-behavioral therapy (CBT) approaches typically dependent on retrospective self-report and giving patients a tailored tool that will help them intervene when they need help the most.

## Method

### Specific aims genomics and microbiota

In 1000 individuals with BN/BED, we will,

Aim 1: Contribute genomic data to the next genome-wide association study (GWAS) conducted by the Eating Disorders Working Group of the Psychiatric Genomics Consortium (PGC-ED) of BN/BED.

Aim 2: Comprehensively characterize the biogeography of the human microbiome using high-throughput sequencing of the microbial 16S rRNA gene and shallow shotgun sequencing.

Aim 3: Employ novel and develop new analytic methods to integrate GWAS, gut microbiota, and phenotypic data that will result in predictive algorithms that index risk, course of illness, severity, disordered eating episodes, and treatment response.

### Specific aims digital longitudinal phenotyping

Aim 1: Conduct longitudinal deep phenotyping of 1000 individuals with BN/BED using *Recovery Record* and Apple Watch.

Aim 2: Predict the occurrence of binge eating and purging (vomiting) episodes in individuals with BN/BED using passive sensor data.

Aim 3: Test theoretically derived regulatory models of binge eating and purging behaviors as reflected in differences in temporal patterns.

Aim 4: Refine our capacity to predict binge and purge episodes by augmenting passive data with contextual factors collected by *Recovery Record*.

### Participants

We are recruiting 1000 individuals with BN or BED.

### Inclusion criteria

1. Currently meets Diagnostic and Statistical Manual for Mental Disorders -5th Edition (DSM-5 [27]) criteria for BN or BED (confirmed via validated questionnaire in screening instrument—see Measures)
2. Resident of US
3. All sexes
4. Age 18–45 years
5. Reads, speaks English
6. Existing iPhone user with iPhone 5 or later
7. Willing/able to wear Apple Watch for entire study period
8. Willing/able to use *Recovery Record* for the entire study period
9. Provides informed consent to have activity and self-reported *Recovery Record* data harvested
10. Ambulatory.

### Exclusion criteria

1. Currently pregnant or breastfeeding
2. Bariatric surgery due to the impact on eating patterns, including the following: (Roux-en-Y gastric bypass, laparoscopic adjustable gastric banding, sleeve gastrectomy, duodenal switch with biliopancreatic diversion, gastric balloon, AspireAssist)
3. Current use of hormone therapy
4. Inpatient treatment or hospitalization for eating disorders in the 2-weeks prior to study enrollment
5. Suicidality at screening
6. Antibiotic or probiotic use in the past 30 days (related to fecal sampling).

## Recruitment

**Fig. 1:**
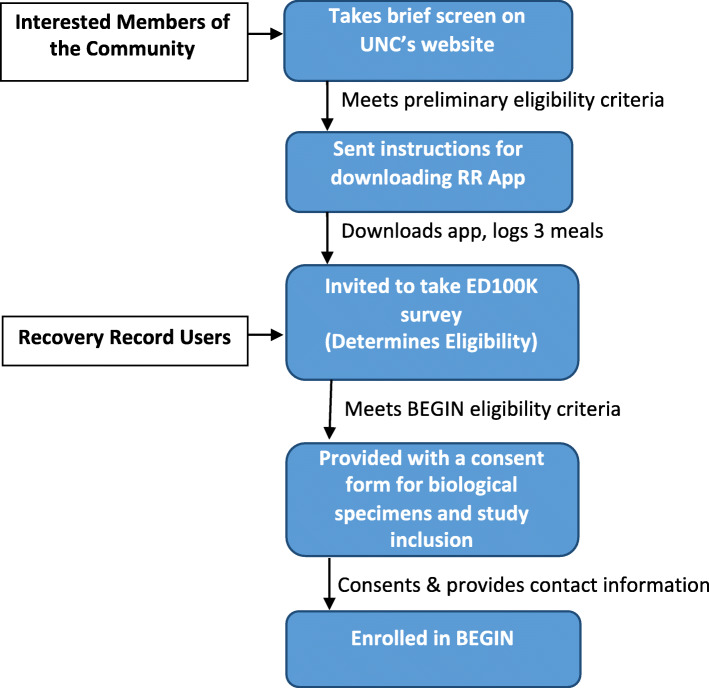
BEGIN study recruitment and sampling flow

We are recruiting cases nationally from diverse geographical, socioeconomic, racial, and ethnic backgrounds via *Recovery Record*, social media and National Eating Disorders Association. Specifically, we launch tweets and Facebook posts that direct potential participants to the BEGIN url https://www.med.unc.edu/psych/eatingdisorders/research/participate-in-a-study/begin-study/ where they can take a preliminary screen. In addition, *Recovery Record* pushes notifications about BEGIN to users. Recruitment flow is detailed in Fig. 1.

### Procedure

Informed consent is obtained digitally via the *Recovery Record* app. Participants complete an eating disorders diagnostic questionnaire. Those who screen case positive and meet all inclusion criteria are offered the opportunity to participate in the full study (with a second digital informed consent). All responses to questionnaires are encrypted and sent to a secure research server at the UNC Sheps Center for Health Services Research using secure transfer methodologies, who compile and house the data in servers specifically designed for Protected Health Information. Data are de-identified (Sheps Center maintains the key to match records). Study data from *Recovery Record* and the Apple Watch are maintained by *Recovery Record* and only includes passive and active sources necessary for analyses, minimizing exposure of protected health information. To ensure that a high level of security is maintained, data transfer from *Recovery Record* occurs with end-to-end encryption and authentication protocols. Records are only identified with a second study number that can be linked using the data from the Sheps Center. Eligible participants are mailed a package containing a description of the study, saliva collection kit, microbiome collection kit, and an Apple Watch. Saliva kits are returned directly to RUCDR Infinite Biologics where they are stored awaiting DNA extraction and genotyping. In Phase 1, participants returned microbiome kits to uBiome for sequencing; in Phase 2, kits are returned to the Carroll lab. Barcodes ensure accurate identification and coordination with phenotypic data. After enrollment and completion of the baseline survey, participants use the Apple Watches and *Recovery Record* for 30 days and complete midpoint and end-of-study surveys at 14 days and 30 days post-enrollment, respectively, to track progress of eating disorder pathology, including binge eating and purging behaviors.

### Measures

#### Deep Phenotyping

Using the *Recovery Record* app and the Apple Watch over a 30-day period for each individual, we conduct active and passive data capture to fully characterize disordered eating behaviors, physical activity, nutrition, gastrointestinal distress, sleep, and heart rate. This generates exceptional data to enable deep characterization of the course of BN/BED. We expect that the likelihood of an event (i.e., binge/purge) will decrease over the course of 30-days and build this expectation into our statistical models. We further expect that although the likelihood of events will change over time, the dynamics of the events will not. These data can be broken down into four categories. First, self-report questionnaires are collected consisting of scales well established to relate to BN and BED (see Self-report questionnaires), measured prior to enrollment or three times across the study. Second, stratified sample intensive measurements consisting of daily mood and meal records are measured 6 times daily. Third, event contingent intensive measurements ask participants to log binge and purge episodes. Finally, continuous passive data collection captures real-time physiological and movement data. These different data will be integrated through multilevel modeling and systems continuous time modeling procedures [28, 29].

#### Active data collection

##### Self-report questionnaires

All BEGIN study participants are screened for eligibility and consented using the *Recovery Record* iPhone app, which is free for users to download and is HIPAA compliant (www.recoveryrecord.com). All questionnaires are completed from within the *Recovery Record* app.

##### ED100K [30]

The ED100K questionnaire is a self-report, eating disorders assessment based on the Structured Clinical Interview for DSM-5, Eating Disorders Module, administered prior to enrollment. Items assess DSM-5 criteria for anorexia nervosa, BN, BED, and other specified feeding and eating disorders. The ED100K-v1 was found to be a valid measure of eating disorders and behaviors [30]. Positive predictive values indicating that among those who had a positive screening test, anorexia nervosa Criterion B, Criterion C, and binge eating ranged from 88 to 100%. Among women who had a negative screen, the probability of not having these criteria or behaviors ranged from 72 to 100%. The correlation between questionnaire and interview for lowest illness-related BMI was *r* = 0.91.

##### Eating disorders examination questionnaire (EDE-Q) [31]

The EDE-Q is a widely used, validated questionnaire capturing eating disorders pathology, including the frequency and severity of binge episodes. The EDE-Q is administered at baseline, midpoint, and endpoint of the 30-day period.

##### The Patient Health Questionnaire (PHQ-9) [32]

Is a 9-item, self-administered version of the PRIME-MD diagnostic instrument for common mental disorders. The nine items are based on the nine DSM-IV criteria for major depressive disorder and are scored as “0” (not at all) to “3” (nearly every day). The PHQ-9 has been found to be a reliable and valid measure of depression severity. The PHQ-9 is administered at baseline, midpoint, and endpoint of the 30-day period.

##### The Generalized Anxiety Disorder 7 (GAD-7) [33]

Is a 7-item, self-report questionnaire to screen for generalized anxiety disorder. Each symptom is scored on a 3-point scale: “not at all” (0), “several days” (1), or “more than half the days” (2). Items are then summed to create a symptom severity score. The GAD-7 is a reliable and valid measure of anxiety. The GAD-7 is administered at baseline, midpoint, and endpoint of the 30-day period.

##### ADHD self-report scale (ASRS) [34]

Is an 18-item questionnaire that assess symptoms associated with attention-deficit/hyperactivity disorder. Items are scored on a 5-point scale. The assessment has high internal consistency and validity [35]. The ASRS is administered at baseline.

##### Rome III [36]

To assess adult GI symptoms of the stomach and intestines, the relevant section (items 17–67) of the ROME III is administered at baseline.

#### Stratified sampled intensive measurements

##### Daily mood and meal records

These data are collected inside the *Recovery Record* iPhone app that primarily targets adherence to meal monitoring tasks. Participants are prompted with a push notification six times per day corresponding to meal and snack times to complete an evidence-based CBT-style question set (what was eaten, with whom, where, and what behaviors were used) in addition to optional symptom-focused questions including current emotional state, urges to engage in eating disorder behaviors, sleeping patterns, hunger levels, gastrointestinal problems, and intrusive thoughts.

#### Event contingent intensive measurements

##### Binge and purge records

**Fig. 2:**
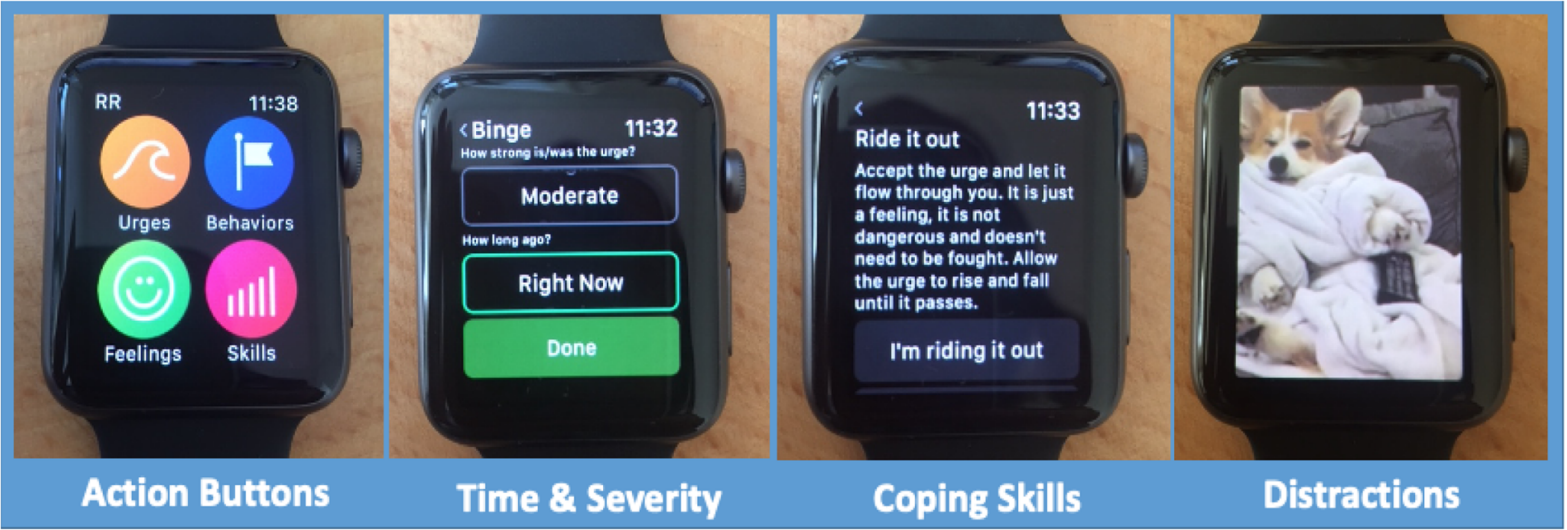
Recovery Record for Apple Watch screen examples. Image Action Buttons include icons created and owned by Recovery Record, Inc. (J. Tregarthen, author, CEO). Image Distractions features *Relaxed Corgi GIF* uploaded by GIPHY, 27 June 2016, https://giphy.com/gifs/7Y66VN3rtkPtu. These images were made available for the purpose of this research per our subcontract agreement with Recovery Record, Inc. (J. Tregarthen, Principal Investigator and author). The image titled Distractions features *Relaxed Corgi GIF* uploaded by GIPHY, 27 June. 2016, https://giphy.com/gifs/7Y66VN3rtkPtu. The GIF was accessed utilizing the Recovery Record, Inc. GIPHY account and made available under a license agreement between Recovery Record, Inc. and GIPHY

Participants are instructed to launch the *Recovery Record* Apple Watch app if they have experienced a binge or purge episode (Fig. 2). Action buttons are used to quickly identify the relevant symptom and how long ago it occurred, with response options in five-minute increments ranging from “Right now” to “30 mins ago”. If an urge to engage in a behavior is identified, participants are additionally asked to rate the urge strength with response options: “Not at all”, “Slight”, “Moderate”, “Strong”, and “Overbearing”. Actively monitored mood, meal, binge and purge records and their respective timestamps are collected on the Recovery Record platform and shared with the research team via encrypted authenticated TLS. Ecological momentary assessment-based logging has shown moderate to strong concordance with retrospective self-report of binge eating and purging [37].

#### Continuous passive data collection

##### Apple watch

The number and timing of the steps (physical activity) as well as 5-min epoch heart rate are passively collected for each study participant using the Apple Watch and harvested by the *Recovery Record* app using Apple’s Application Program Interface (API). The Apple Watch activates the sensor approximately every 5 min to record heart rate based on 100 Hz using photo plethysmography. Built in signal processing algorithms are used to aggregate measurements to approximately 5-min intervals, a rate consistent with current methodological guidelines (e.g., Berntson [38]). To minimize data loss, these variables are uploaded to the *Recovery Record* server each time the *Recovery Record* app is opened on the iPhone while the Apple Watch is nearby, or at least once per day.

#### Biological sampling

##### Saliva sampling and genotyping

Saliva samples are collected with RUCDR Infinite Biologics saliva collection kits. GWAS profiling will be performed together with additional samples collected by the PGC-ED using the optimal platform at the time of genotyping, most likely a version of the Illumina Global Screening Array (GSA).

##### Fecal sampling and sequencing

In Phase 1, as recipients of a scientific in-kind grant from the now defunct company uBiome, we collected stool samples. ***uBiome*** comprehensively characterized the biogeography of the human microbiome using high-throughput sequencing of the microbial 16S rRNA gene and released all data to UNC for analysis. After their company dissolved in Oct 2019, all processing transferred to the Carroll Lab at UNC (I. Carroll, Director). In order to obtain high-resolution taxonomic and functional microbiome data, we will perform whole genome shotgun sequencing. Raw sequence data will be quality filtered and trimmed to remove bases with Phred quality scores less than 20. Downstream bioinformatics analysis will consist of: i) taxonomic composition; ii) functional composition; iii) alpha diversity (as measured by absolute numbers of sequence variants and the Shannon index of diversity) and (quantified by Bray Curtis and UniFrac metrics); and iv) computing descriptive statistics and identifying groups within the data, as well as performing statistical analyses between subgroups using additional metadata, where available [39]. Since the sequencing technology and bioinformatics tools are rapidly advancing, we will utilize the most suitable methods and tools available at the time of analysis.

### Planned data analysis

#### Genomics and microbiota aims

We will combine BEGIN samples with other samples in the PGC-ED for meta-analysis. We will conduct cross-disorder analyses to identify loci that cut across diagnostic categories by leveraging existing high-quality results for anorexia nervosa, major depressive disorder, schizophrenia, bipolar disorder, and other psychiatric and metabolic phenotypes. We will use advanced methods [40] to compute SNP heritabilities and genetic correlations across psychiatric and metabolic traits. We will calculate metabolic and psychiatric trait & disorder polygenic scores (PGS) using PRSice, (http://www.prsice.info). A leave-one-sample-out process will be carried out to calculate BN/BED PGSs. The calculated PGS will be the weighted numbers of risk alleles carried by each case and control. This aim will illuminate, from a fundamental perspective, the genetic architecture of BN/BED and its relation to other psychiatric disorders and metabolic conditions.

We will compare taxonomic composition and diversity of the gut microbiota for BEGIN participants, compare BN with BED, and both to a reference control panel. We will control for multiple covariates in all analyses (e.g., obesity).

We can now rapidly do GWAS on multiple phenotypes – e.g., GWAS for 22 K transcriptomic, 8 K proteomic, or 1 K metabolomic measures. (1) We will adapt and extend these methods to evaluate host genomic-microbiota interactions by conducting ~ 15 K GWAS for species-level microbial measures while controlling for multiple comparisons. (2) We will generate microbiome “modules”, clusters of species with high intra-group correlations and low inter-group correlations. We will then do a GWAS for these modules. (3) For all analyses, we will pay particular attention to the genomic regions highlighted in the prior literature (e.g., MHC, autoimmunity, gut barrier, inflammatory bowel disease). (4) We will utilize publicly available databases of summary statistics across a range of psychiatric, personality, metabolic, and physical activity phenotypes and employ both trait-specific polygenic scores (PGS) and multi-polygenic scores (MPS) to predict outcomes. We will use a novel MPS approach developed by collaborator Breen and colleagues [41], that exploits genetic correlations between the outcome trait and a multitude of traits by using the joint predictive power of multiple polygenic scores in one regression model. We will select relevant GWAS from a centralized repository of summary statistics to predict BN, BED, severity, treatment outcome. Using repeated cross-validation, we will train and validate the prediction models using elastic net regularized regression, which is a multiple regression model suited to deal with a large number of correlated predictors while preventing overfitting [42]. We will then add microbiota and phenotyping variables into the model to improve predictive accuracy.

#### Digital longitudinal phenotyping aims

Our dynamic systems approach capitalizes on a combination of the passive and active data collection to address all three of the longitudinal phenotyping aims. Each stable state can be thought of as having homeostatic properties that are reflected in associations between different levels of derivatives (i.e., change in value with respect to time). For example, the relationship between changes in heart rate from one moment to the next and values of heart rate at the previous moment characterize how heart rate fluctuates homeostatically about a “set point.” This set point represents the heart rate value to which the individual’s body returns when the person is at rest [43]. Not only does this association characterize the homeostatic heart rate value itself but also the rate of return to the set point when a person’s heart rate is perturbed (i.e., experiencing distress prior to a binge/purge episode, physical load creating during exercise, etc.). Higher order derivatives and accounting for more variables simultaneously allows for testing more complex homeostatic patterns (e.g., cycles), while including this concept of rate of return to set point (i.e., systemic stability).

Aim 2 will be tested by first depicting the dynamics that lead up to a binge or purge event in a multilevel model. Aim 3 will be addressed by depicting the dynamics once a binge or purge event has occurred. In this case, analyses will focus on the 2 h after binge/purge events (but not within an hour of a future event), again modeling changes in heart rate and steps as a function of current levels in heart rate and steps. Aim 4 will require depicting each instance in time in terms of risk for being in one of the temporal states associated with subsequent binge eating and/or purging. To do so, we will utilize the posterior probabilities from a latent mixture model where each pattern is differentiated by associations amongst different levels of change. Mixture modeling is a taxonomic approach where timepoints within and between individuals can be grouped together as a function of a model. In this case, the model will differentiate groups of data as a function of the dynamic properties.

To help ensure reproducibility, the sample will be split in half with each half used as confirmation on the other half generating competing models. Under large data circumstances such as these, rather than power, the primary concern is a combination of overfitting and gaining a proper gauge of an effect. Generating competing models allows each of the samples to function as confirmation of the other with the better fitting model on both samples providing the more generalizable solution.

## Discussion

As a multi-pronged investigation, BEGIN will have broad impact across various dimensions in the eating disorders field. First, in the biological domain, BEGIN will allow us to identify genetic and gut microbiota contributors to disorder risk and maintenance and identify genomic, enteric microbes, and behavioral predictors of outcome. Second, in the behavioral domain, BEGIN will allow us to build algorithms that predict behavioral events (e.g., impending binges or purges) to enable real-time intervention via wearable technology.

We intend this to be a transformative study in the field of eating disorders. Through deep longitudinal phenotyping via the Apple Watch, we designed BEGIN to rapidly accelerate progress toward personalized precision medicine for BN and BED. Advances in eating disorders treatment have been slow and incremental. In our Agency for Healthcare Quality and Research review of treatments for BED [44], we noted that the evidence base was challenged by small samples and single studies introducing small variations on core therapeutic approaches with little or no additive efficacy. Wearable sensors, such as the Apple Watch with the adapted *Recovery Record* app, offer us the opportunity to develop a transformative improvement in BN and BED treatment. BN and BED are model disorders with discrete and measurable pathognomonic unhealthy behaviors. By applying dynamical systems models to the passive and active data that we collect, we will bypass historical one-size-fits-all CBT interventions for BN and BED and immediately enter the era of personalized interventions for eating disorders. Not only will we be able to build models that predict binge and purge episodes within the acute phase of the illness, but personalized extensions of these models will allow us to identify and alert individuals to impending slips and relapses after recovery.

Although traditional CBT interventions that rely on in-session retrospective recall will never be entirely obsolete, we expect that the just-in-time approach afforded by our dynamical systems models will render ours a central feature in the future treatment of BN/BED. Results from BEGIN will set the stage for subsequent studies in which we will have achieved the ability to discriminate across types of events (e.g., exercise, meal, binge, purge) that will allow us to build in accurate push notifications when an individual’s passive and active data signal an impending binge or purge—truly tailoring treatment and delivering it in-the-moment. Moreover, *Recovery Record* already has a clinician interface, and we predict that we will be able to incorporate our models into provider interfaces such that clinicians will be able to view and interact with the alerts that emerge, thus supporting the provision of data-informed care.

Ultimately, we foresee that this study will advance both cognitive-behavioral approaches to understanding and treating eating disorders and dynamical systems theory of behavior change to incorporate both intensive longitudinal behavioral and physiological data. Although our focus is on eating disorders, we intend our models to be readily adaptable for other psychiatric (and somatic) conditions that have identifiable measurable indices in order to usher us more rapidly toward individualized interventions that attend to the psychology, the biology, and the dynamic environment of the individual.

## Data Availability

Our liberal data and analysis sharing principles will make genomic, microbiota, and phenotypic data and scripts widely available for access by other scientists to maximize utility of our investigation. The datasets generated and/or analyzed during the current study will be available in the National Data Archive (https://nda.nih.gov/) and on Open Science Framework (https://osf.io/). DOI 10.17605/OSF.IO/KJ7WR. DNA samples will be available from the NIMH Repository and Genomics Resource (https://www.nimhgenetics.org/order-biosamples/how-to-order-biosamples).

## Abbreviations

ADHD: Attention deficit hyperactivity disorder
ANS: Autonomic nervous system
ASRS: ADHD Self-Report Scale
API: Application programming interface
BED: Binge-eating disorder
BEGIN: Binge Eating Genetics Initiative
BN: Bulimia nervosa
CBT: Cognitive-behavioral therapy
DNA: Deoxyribonucleic acid
DSM IV: Diagnostic and Statistical Manual of Mental Disorders 4th Edition
DSM 5: Diagnostic and Statistical Manual of Mental Disorders 5th Edition
ED100K: Eating Disorders 100,000 Questionnaire
EDE-Q: Eating Disorders Examination-Questionnaire
GAD-7: Generalized Anxiety Disorder-7
GSA: Global Screening Array
GWAS: Genome-wide association study
HIPAA: Health Insurance Portability and Accountability Act
MPS: Multi-polygenic score
PGC-ED: Eating Disorders Working Group of the Psychiatric Genomics Consortium
PGS: Polygenic score
PHQ-9: Patient Health Questonnaire-9
RUCDR: Rutgers University Cell and DNA Repository
SNP: Single nucleotide polymorphism
UNC: University of North Carolina
